# Animal models of chemotherapy‐induced peripheral neuropathy for hematological malignancies: A review

**DOI:** 10.1002/ibra.12086

**Published:** 2022-12-22

**Authors:** Xiaoli Lv, Yingwei Mao, Song Cao, Yonghuai Feng

**Affiliations:** ^1^ Department of Hematology Affiliated Hospital of Zunyi Medical University Zunyi Guizhou China; ^2^ Department of Biology Penn State University University Park Pennsylvania USA; ^3^ Department of Anesthesiology Affiliated Hospital of Zunyi Medical University Zunyi China; ^4^ Department of Pain Medicine Affiliated Hospital of Zunyi Medical University Zunyi China

**Keywords:** animal models, chemotherapy, chemotherapy‐induced peripheral neuropathy, multiple myeloma

## Abstract

Chemotherapy is one of the main treatments for hematologic malignancies. However, chemotherapy‐induced peripheral neuropathy (CIPN) is one of the most common long‐term toxic reactions in chemotherapy, and the occurrence of CIPN affects patients’ quality of life and can cause interruption of chemotherapy in severe cases, thus reducing the efficacy of chemotherapy. We currently summarize the existing CIPN animal models, including the characteristics of several common animal models such as bortezomib‐induced peripheral neuropathy, vincristine‐induced peripheral neuropathy, and oxaliplatin‐induced peripheral neuropathy. It was found that CIPN may lead to behavioral, histopathological, and neurophysiological changes inducing peripheral neuropathy. However, the mechanism of CIPN has not been fully elucidated, especially the prevention and treatment protocols need to be improved. Therefore, this review article summarizes the progress of research on CIPN animal models and the possible mechanisms and treatment of CIPN.

## Graphical abstract

CIPN from different sites of the nervous system. Animal models (BIPN, VIPN, OIPN, and TIPN) were used to construct PN induced by four kinds of chemotherapy drugs in hematological malignancies. The neurotoxicity of CIPN is mainly concentrated in the peripheral nervous system, including the DRG, axons and axonal components (myelin, mitochondria, microtubules, and blood vessels), and nerve terminal. In addition, CIPN can be treated with therapies such as Western medicine and traditional Chinese medicine to relieve pain. CIPN, chemotherapy‐induced peripheral neuropathy; PN, peripheral neuropathy; BIPN, bortezomib‐induced PN; VIPN, vincristine‐induced PN; OIPN, oxaliplatin‐induced PN; TIPN, thalidomide‐induced PN; DRG, dorsal root ganglion.

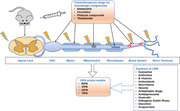

## 1 INTRODUCTION

Chemotherapy‐induced peripheral neuropathy (CIPN) is a common drug dose‐limiting adverse reaction in cancer patients during chemotherapy. It is progressive, persistent and irreversible,^1^, ^2^ mainly manifested as sensory and motor abnormalities, as well as autonomic dysfunction, sensory ataxia, limb weakness, numbness, tingling, and burning sensation, accompanied by loss of intraepidermal nerves of fibers (IENFs).^3^ The numbness and tingling are the earliest, and the glove‐like distribution usually begins at the distal ends of the fingers and toes. Neuropathic pain, which is not only complicated in mechanism but also difficult to treat,^4^, ^5^ is one of the most serious symptoms caused by CIPN. Symptoms may last for months or even years as the disease progresses toward the proximal end.^6^ With the increasing incidence of malignancies year by year, chemotherapy drugs are used more and more widely as first‐line antitumor drugs. According to statistics, about 30%–40% of chemotherapy patients will suffer from PN.^2^

**Figure 1:**
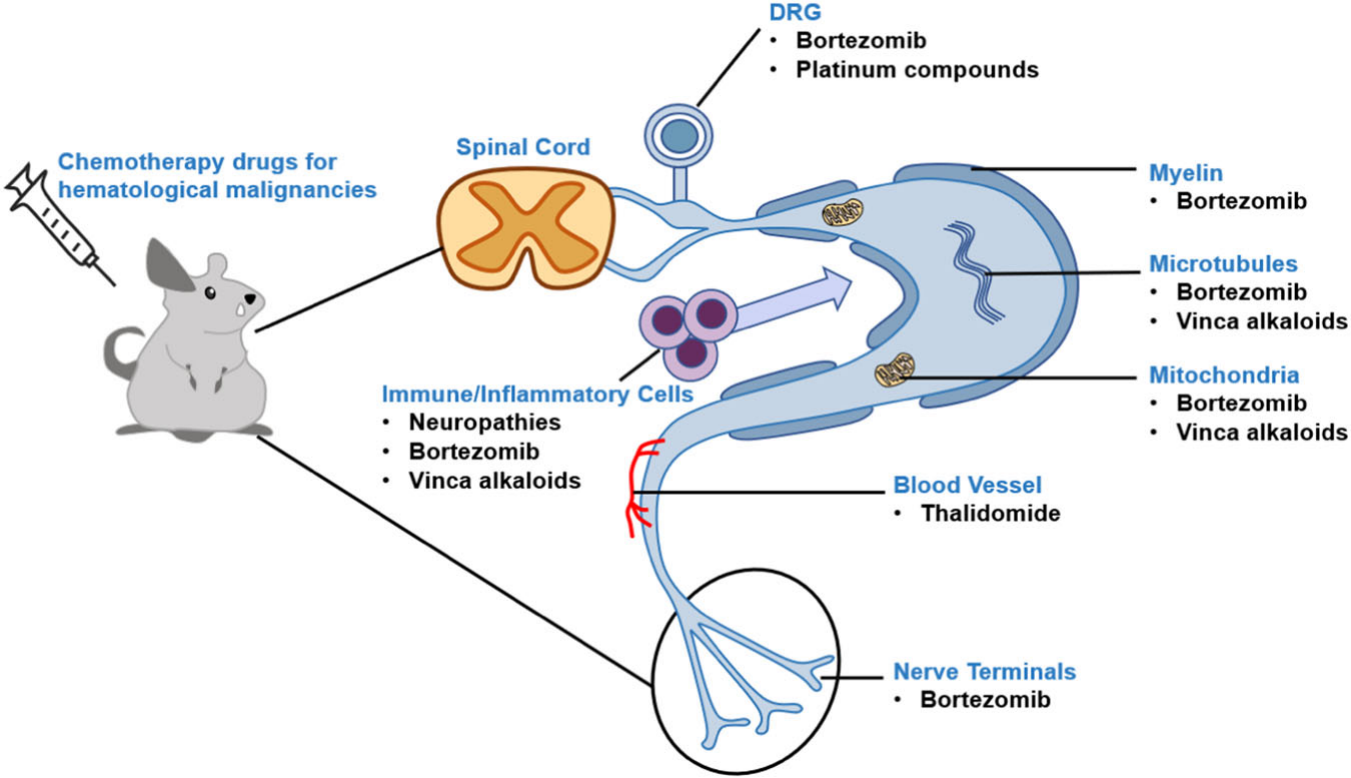
Multiple targets of chemotherapy in CIPN animal models. Chemotherapy drugs induce neurotoxicity for hematologic malignancies predominantly in the peripheral nervous system, namely the DRG, axons and axonal components (myelin sheath, microtubules, mitochondria, and blood vessels), and nerve terminals. In addition, PN can also be induced by increasing neuroinflammation. CIPN, chemotherapy‐induced peripheral neuropathy; DRG, dorsal root ganglion; PN, peripheral neuropathy. [Color figure can be viewed at wileyonlinelibrary.com]

In the field of hematological malignancies, chemotherapy is still an important treatment. Drugs associated with CIPN in hematological malignancies include proteasome inhibitors (such as bortezomib [BTZ]), vinca alkaloids (such as vincristine), cytotoxic antitumor agents (such as cisplatin and oxaliplatin [OXP]), and immunomodulators (such as thalidomide),^2^ which adversely affect the peripheral nervous system through the different mechanisms summarized in Figure 1. As the number of malignancies continues to increase, more and more patients receive chemotherapy, and the accompanying CIPN also increases. CIPN not only increases patients’ physical and mental pain, hinders the successful completion of chemotherapy but also generates a high economic burden for patients. Therefore, how to reduce CIPN in patients is particularly important. The establishment of an experimental animal model with uniform and standardized standards that can objectively quantify CIPN can lay the foundation for further prevention and treatment. This article briefly reviews the mechanism of CIPN and the research progress of existing animal models.

## 2 DRUGS USED FOR CIPN MODELING IN ANIMALS WITH HEMATOLOGICAL MALIGNANCIES

A variety of antihematological malignancies chemotherapy drugs can cause postchemotherapy neuralgia. The drugs selected for the experimental modeling of CIPN are usually screened according to the extensive clinical use of the drug and the incidence of PN caused by injection. Currently, proteasome inhibitors, immunomodulators, and vinca alkaloids are commonly used to treat multiple myeloma, lymphoma, and leukemia, and platinum‐based compounds are commonly used to treat lymphoma.^7^ Development of CIPN during antitumor therapy can result in dose reduction, delay, or discontinuation of treatment, and the persistence of symptoms after treatment can adversely affect the patient's quality of life.^8^

## 3 CIPN MODEL

### 3.1 BTZ‐induced CIPN model

BTZ is the first proteasome inhibitor to enter clinical use. After cell‐cycle inhibition, BTZ exerts antitumor effects by inhibiting the proteasome ubiquitination mechanism through its unique reversible binding to the 26S protease subunit,^9^ and ultimately leading to apoptosis.^10^, ^11^ It is mainly used to treat multiple myeloma, and can also be used in combination with other drugs to treat other hematological and solid tumors.^12^ Although BTZ has shown potent antitumor activity in patients, its induced PN is one of the main reasons why patients discontinue BTZ. Since the pathogenesis of bortezomib‐induced PN (BIPN) caused by BTZ treatment has not been fully elucidated, the understanding of BIPN is mainly based on the results of animal models.

Cavaletti et al.^13^ conducted preclinical studies on BTZ‐induced neurotoxicity and established a rat model of BIPN for the first time. Seventy‐two adult female Wistar rats were selected and divided into control group and BTZ group. The low dose intensity (0.08 mg/kg/d, 2q7d ×4) to the maximum tolerated dose (0.20 mg/kg/d, 3q7d ×4) were used to compare the effects of long‐term administration on the peripheral nervous system and spinal cord of rats. The results showed that the 2q7d and 3q7d doses of 0.08, 0.15, and 0.20 mg/kg were well tolerated in rats, but the rats died at 0.30 mg/kg. The caudal nerve conduction velocity (SNCV) was only significant in the 0.20 mg/kg group on 2q7d, and decreased significantly from 14 to 42 days. Mild to moderate sciatic nerve lesions occurred in rats with 0.20 mg/kg (two regimens), mainly in Schwann cells and myelin sheath, and axonal degeneration was also observed. Similarly, BTZ‐induced changes were also observed in the dorsal root ganglion (DRG) at the same dose, and the satellite cells were severely damaged and intracytoplasmic vacuoles occurred, and the BIPN model was considered successful. It also illustrates the importance of chemotherapy dose control on postchemotherapy severity and mortality. After that, many researchers also adopted the rat model in vivo and successfully replicated the peripheral neurotoxicity characteristics of the drug.^14^, ^15^, ^16^

Bruna et al.^17^ also replicated BIPN in mice. Twenty female OF1 mice were selected and subcutaneously administered BTZ at 0.8, 1 mg/kg 2q7d, and 1 mg/kg 3q7d for 6 weeks. The results showed that a dose of 1 mg/kg 2q7d was well tolerated, and only one mouse died in the 2nd week after the third BTZ treatment, which induced mild to moderate sensory neuropathy. Histopathology showed that myelinated fibers and unmyelinated fibers were slightly reduced, and further studies found that the structure of axons and myelin sheaths also changed, indicating peripheral nerve damage, which was consistent with the characteristics of PN. This model could be a useful model for elucidating potential mechanisms of BTZ neurotoxicity and testing the safety of neuroprotective treatments or new BTZ dosing schedules. Although bortezomib‐induced neuralgia is clinically easy to diagnose and reliable models are available, its pathophysiological mechanisms remain partially unclear. Immunomodulation has been proposed as a possible therapeutic approach for BIPN. Thus, Carozzi et al.^18^ used immunocompetent and deficient BALB/C mice; BTZ was given at 0.8 mg/kg twice a week for 4 weeks via the tail vein. No mice died during the experiment, and body weight measurements showed that the weight loss after the injection continued until the end of the experiment. As reported in previous studies, BTZ treatment induced marked lesions in the spinal cord, dorsal root, DRG, sciatic nerve, and coccygeal nerve, manifested by significant reductions in SNCV, amplitude, and mechanical threshold in mice.^19^ Histopathological observations confirmed that BTZ‐induced DRG lesions could cause sciatic nerve axonal degeneration, and an animal model of PN in humans was successfully established, which was almost similar to previous experimental studies on rats.^13^, ^19^ The study also confirmed that immune response may not be a key factor in the development of BTZ‐induced morphological and functional impairment in the peripheral nervous system.

Since the underlying mechanisms of BIPN are unclear, although it has been shown that they include direct DRG toxicity and nerve fiber damage, the severity of BIPN used to treat multiple myeloma is significantly different from that of solid tumors. This incomplete knowledge and apparent clinical need suggest that the importance of a deeper understanding of the pathogenesis of BIPN also requires the use of innovative animal models encompassing the effects of multiple myeloma and BTZ.^20^ Several of these animal studies evaluated the effects of BTZ on the peripheral nervous system. For example, studies on immunocompetent C57BL/6 mice have extensively studied the characteristics of animal BIPN using electrophysiological, behavioral, and pathological approaches to successfully simulate the BIPN model.^21^, ^22^ However, no animal studies have been performed on animals with multiple myeloma. Therefore, the complex nerve damage typical of BTZ therapy in patients with multiple myeloma is ignored. Next, Meregalli et al.^23^ conducted a study using immunodeficient SCID mice. RPMI8266 human multiple myeloma cells were injected subcutaneously, and BTZ 1 mg/kg was administered via the tail vein once a week for 5 consecutive weeks to successfully establish a neuralgia model. By the end of the study, BIPN was evident in animals, with DRG toxicity and nerve fiber damage in the BTZ group, myeloma + BTZ mice, and mild lesions in the myeloma group. This tumor‐bearing multiple myeloma animal‐based model more reliably reproduces the clinical setting. Therefore, it is more suitable to study its pathogenesis. A study found that subcutaneous (SC) BTZ resulted in a lower incidence of PN compared with intravenous (IV).^24^ In addition, several other studies have shown that once a week SC BTZ, compared with twice a week SC, once a week IV, twice a week IV, the incidence of BIPN decreased, and the efficacy was not significantly different from other groups.^25^, ^26^, ^27^ Despite the low neurotoxicity of SC injections, BIPN remains a serious, potentially dose‐limiting side effect.^28^ In conclusion, it is important to establish an in vivo model to further elucidate the relevant mechanisms of BIPN and help improve the clinical treatment of BIPN patients (Table 1). Furthermore, it may be a useful model for evaluating neuroprotective agents designed to ameliorate or restore BIPN and to inform the safety of new BTZ dosing regimens.

**Table 1:** Summary of animal models of CIPN induced by chemotherapeutic agents in hematologic malignancies

Chemotherapy agent	Dosing regime	Animals	Findings	Possible mechanism	Reference
Bortezomib	iv, 0.08, 0.15, 0.2, and 0.3 mg/kg, 2q7d or 3q7d,4 weeks	Wistar rats	Sciatic nerve degeneration, DRG morphologic changes, and decreased SNCV.	Targeted injury to DRG cells, neurohypophysis, and nerve axons.	Cavaletti et al.^13^
	ip, 0.2 mg/kg, d1‐5	SD rats	Mechanical, cold allodynia, and decreased IENF density.	Mitotoxicity and mitochondrial dysfunction.	Zheng et al.^14^
	ip, 0.05, 0.1, 0.15, and 0.2 mg/kg, d1,3,5,7	SD rats	Mechanical hyperalgesia and spinal WDR neurons showed increased firing and persistent after‐discharges.	Glutamate transporter protein dysfunction.	Robinson et al.^15^
	sc, 0.2 mg/kg, 3q7d, 8 weeks	Wistar rats	Mechanical hyperalgesia and decreased SNCV.	Inflammatory response.	Zhao et al.^16^
	sc, 0.8,1 mg/kg, 2q7d or 3q7d, 6 weeks	OF1 mice	Sciatic nerve degeneration, IENF density, SNCV, and SNAP were decreased.	Targeted injury to DRG cells, neurohypophysis, and nerve axons.	Bruna et al.^17^
	iv, 0.4 and 0.8 mg/kg, 2q7d, 4 weeks	BALB/c mice	Sciatic nerve degeneration, DRG morphologic changes, decreased SNCV and SNAP.	Targeted injury to DRG cells and nerve axons.	Carozzi et al.^19^
	iv, 0.8 mg/kg, 2q7d, 4 weeks	BALB/c mice	Mechanical nociception, spinal WDR neurons showed an increase, SNCV and SNAP decreased.	Targeted injury to DRG cells and nerve axons; mitochondrial and endoplasmic reticulum damage.	Carozzi et al.^18^
	ip, 1 mg/kg	C57BL/6 mice	Mechanical and cold hyperalgesia.	TRPA1 channel activation.	Tonello et al.^21^
	0.4 mg/kg, 3q7d, 4 weeks	C57BL/6 mice	Mechanical, thermal allodynia, DRG morphologic changes and spinal cord GFAP increased.	Immune function regulators PKs are involved in neurotoxicity.	Moschetti et al.^22^
	iv, 1 mg/kg, 1q7d, 5 weeks	SCID mice	Morphological changes of DRG.	Targeted injury to DRG cells.	Meregalli et al.^23^
Vincristine	iv, 100 μg/kg/d, 2 weeks	SD rats	Mechanical and thermal hyperalgesia.	Disrupted the microtubule‐dependent function.	Aley et al.^29^
	iv, 100 μg/kg/d, 2 weeks	SD rats	Mechanical hyperalgesia and slowed conduction velocity of sensory neurons.	Abnormal discharge of class C fibers.	Tanner et al.^30^
	iv, 0, 1, 10, 30, 50, and 100 μg/kg/d, 2 weeks	SD rats	Tactile and thermal allodynia;	Changes in microtubule dynamics, upregulation of sodium channels, activation of spinal glutamate receptors, and modulation of opioid‐related receptors.	Nozaki‐Taguchi et al.^31^
	ip, 100 μg/kg, 5q7d, 2 weeks	SD rats	Mechanical and thermal hyperalgesia.	Targeted injury to DRG cells.	Youn et al.^32^
	ip, 0.1 ml/kg/d, 5q7d, 2 weeks	SD rats	Mechanical allodynia.	WDR neurons and NMDA receptors are centrally sensitized.	Park et al.^33^
	ip, 100 μg/kg, 5q7d, 2 weeks	SD rats	Mechanical and cold allodynia.	Oxidative stress and inflammatory response.	Kahng et al.^34^
	ip, 100 μg/kg, 5q7d, 2 weeks	SD rats	Mechanical and cold allodynia; TNF‐α and MPO activity increased.	Inflammatory response.	Kim et al.^35^
	0.5 µl/h, 14 days	SD rats	Mechanical allodynia and central sensitization; and decreased EM‐2 in the spinal cord.	Modulation of opioid‐related receptors.	Yang et al.^36^
	0.1 mg/kg, d1–10	SD rats	Mechanical allodynia and thermal hyperalgesia decreased GABAergic Synaptic Function	Alterations in spinal synaptic plasticity that sustain neurological symptoms	Wang et al.^37^
	ip, 100 μg/kg, d1–10	Wistar rats	Cold allodynia, mechanical, paw heat, and cold tail hyperalgesia.	Oxidative stress and increase in total calcium content.	Kaur et al.^38^
	ip, 75 μg/kg, d1–10	Wistar rats	Mechanical, thermal allodynia and hyperalgesia, and loss of sciatic nerve function.	Oxidative stress, inflammatory response, and increase in total calcium content.	Muthuraman et al.^39^
	ip, 100 μg/kg, 2 weeks	Wistar rats	Sciatic nerve degeneration, mechanical and thermal allodynia; increased TNF‐α and IL‐1β.	Inflammatory response and activation of NF‐κB signaling pathway.	Khalilzadeh et al.^40^
	ip, 100 μg/kg, d1–7	Swiss albino mice	Mechanical, thermal allodynia, and loss of sciatic nerve function.	Oxidative stress and increase in total calcium content.	Babu et al.^41^
	ip, 100 μg/kg, d1–7	Mice	Mechanical hyperalgesia and cold allodynia.	Increase in inflammatory response and oxidative stress.	Linglu et al.^42^
	ip, 100 μg/kg, d1–7	DDY‐strain mice	Mechanical allodynia.	Activation of the noradrenergic system.	Katsuyama et al.^43^
	ip, 100 μg/kg, d1–8	Swiss mice	Sciatic nerve degeneration, mechanical allodynia, decreased DRG and IENF neurons.	Abnormal response of class A and C fibers.	Bessague et al.^44^
	ip, 50 μg/kg, d1–10	BALB/c mice	Mechanical, cold allodynia, and thermal hyperalgesia.	Oxidative stress.	Khan et al.^45^
	ip, 100 μg/kg, 5q7d, 2 weeks	mice	Mechanical allodynia, NK‐1, and SP increased in spinal cord.	Altered synaptic plasticity in the spine, which maintains neurological symptoms.	Lee et al.^46^
	ip, 75 μg/kg, d1–10	BALB/c mice	Sciatic nerve degeneration, mechanical, cold allodynia, and thermal hyperalgesia.	TRP channels, P2Y and MAPK signaling, inflammatory response, and oxidative stress.	Khan et al.^47^
	ip, 75 μg/kg, d1–10	Mice	Mechanical, cold allodynia and thermal hyperalgesia, decreased SNCV and SNAP, increased TNF‐α, IL‐6, and MPO.	Inflammatory response and oxidative stress.	Gong et al.^48^
	ip, 0.5 mg/kg, 5q7d, 2 weeks	C57BL/6 mice	Peripheral neuropathy of the sciatic nerve increased TNF‐α, IL‐1β, IBA‐1, and CD11b.	Inflammatory response.	Zhang et al.^49^
	ip, 0.1 mg/kg, d1–5	Mice	Mechanical allodynia and thermal hyperalgesia increased TNF‐α, IL‐6 and IL‐1β; increased GFAP in the spinal cord.	Altered calcium ion movement in mitochondria	Li et al.^50^
Oxaliplatin	ip, 4 mg/kg, 2q7d, 4 weeks	SD rats	Mechanical and cold hyperalgesia.	Ion channel disorders and central sensitization.	Zhou et al.^51^
	ip, 2.4 mg/kg, 5q7d, 3 weeks	SD rats	Mechanical and cold hyperalgesia, motor dysfunction; and increased GFAP and IBA‐1 in the spinal cord.	TRP channel activation and spinal astrocyte activation.	Hao et al.^52^
	ip, 2.4, 3.2, 4 mg/kg, 2q7d, 4.5weeks	SD rats	Mechanical hyperalgesia and cold allodynia.	Ion channel disorders.	Li et al.^53^
	ip, 2.4 mg/kg, d1–5	SD rats	Mechanical and thermal hyperalgesia	Pain‐related gene expression and signaling pathways such as PPAR are related.	Yang et al.^54^
	ip, 4 mg/kg, 2q7d, 4weeks	SD rats	Mechanical and cold hyperalgesia, decreased sciatic nerve conduction velocity, and increased TNF‐α, IL‐1β, and IL‐6.	Oxidative stress‐mediated NF‐κB activation and inflammatory response.	Zhang et al.^55^
	ip, 0.4 mg/100 g/d, d1–5	SD rats	Mechanical allodynia, increased TNF‐α and IL‐1β.	NF‐κB activation and inflammatory response.	Huang et al.^56^
	ip, 2.5 mg/kg, d1–4	SD rats	Mechanical, cold allodynia, and thermal hyperalgesia, increased TNF‐α and IL‐6.	NF‐κB activation and inflammatory response.	Ni et al.^57^
	ip, 2.5 mg/kg, 5q7d, 3 weeks	SD rats	Mechanical and cold hyperalgesia.	Ion channel disorders	Di Cesare Mannelli et al.^58^
	ip, 4 mg/kg,2q7d,4 weeks	SD rats	Mechanical hyperalgesia, heat hypoalgesia, and cold allodynia.	Ion channel disorders	Liu et al.^59^
	ip, 4 mg/kg, d1,2,5,6	Wistar rats	Cold, thermal hyperalgesia, and motor dysfunction;	Oxidative stress and mitochondrial dysfunction.	Waseem et al.^60^
	ip, 4 mg/kg, 2q7d, 4 weeks	Wistar rats	Mechanical hyperalgesia, cold allodynia, and DRG morphologic changes.	Targeted damage to DRG cells.	Cheng et al.^61^
	ip, 2.4 mg/kg, 5q7d, 2 weeks	Wistar rats	Mechanical and thermal hyperalgesia.	Abnormal excitability of DRG neurons.	Resta et al.^62^
	ip.6 mg/kg	C57BL/6 mice	Mechanical, cold allodynia and increased IL‐1β.	Inflammatory response and activation of glial cells.	Li et al.^63^
	ip, 3 mg/kg, 2, 4, 6, 24, 26, 48, and 72 h	C57BL/6 mice	Mechanical hyperalgesia.	Inflammatory mediators cause the activation of TRP channels and the involvement of lipid signaling pathways.	Rimola et al.^64^
	ip, 10 mg/kg, 1q7d, 4–6 weeks	C57BL/6 mice	Sciatic nerve degeneration, mechanical, cold allodynia, and decreased NCV.	Endoneurial microvascular dysfunction.	Ogihara et al.^65^
	ip, 6 mg/kg	C57BL/6 mice	Sciatic nerve degeneration, mechanical, cold allodynia, decreased NCV, loss of motor coordination, and depression‐like behavior.	Altered monoamine (5‐HT/NA) levels.	Micov et al.^66^
	ip, 6 mg/kg	C57BL/6Babr mice	Mechanical and cold hyperalgesia.	Mitochondrial dysfunction.	Gould et al.^67^
	ip, 10 mg/kg, 1q7d, 3 weeks	BALB/c mice	Mechanical, cold hyperalgesia, and decreased IENF density.	Mitochondrial dysfunction.	Toyama et al.^68^
	ip, 5 mg/kg, d1–3	Swiss mice	Cold hyperalgesia and decreased tactile sensation.	Involvement of the renin‐angiotensin system.	Bouchenaki et al.^69^
Thalidomide	po, 10.5 mg/kg/d, 2 weeks	SD rats	Sciatic nerve degeneration.	‐	Liu et al.^70^

Abbreviations: IBA‐1, ionized calcium‐binding adapter molecule‐1; DRG, dorsal root ganglion; EM‐2, endomorphin‐2; GFAP, glial fibrillary acid protein; IENF, nerve fibers in the epidermis; ip, intraperitoneal injection; iv, intravenous injection; MAPK, mitogen‐activated protein kinase; MPO, myeloperoxidase; NCV, nerve conduction velocity; NF‐κB, nuclear factor kappa‐B; NK‐1, neurokinin‐1; NMDA, *N*‐methyl‐d‐aspartate; P2Y, purinergic receptor; PKs, prokineticins; po, per os; PPAR, peroxisome proliferator‐activated receptor; SNAP, sensory nerve action potential; SNCV, sensory nerve conduction velocity; TRP, transient receptor potential; TRPA1, transient receptor potential A1; sc, subcutaneous; SP, substance P; WDR, wide dynamic range.

### 3.2 Vincristine‐induced CIPN model

Vincristine is a chemotherapeutic drug belonging to the group of vinca alkaloids, which includes vinblastine and vindesine, and is a highly active cell cycle‐dependent anticancer drug.^71^ It binds to tubulin, causing depolymerization of microtubules in cells undergoing mitosis, cell cycle arrest, and apoptosis, thereby exerting its antitumor effect.^72^, ^73^ It is primarily used to treat several solid tumors and hematological malignancies, including non‐Hodgkin lymphoma and leukemia.^7^ Vincristine is the most neurotoxic of the vinca alkaloids and induces PN in a dose‐dependent manner.^74^, ^75^, ^76^ This further results in decreased morbidity and quality of life for patients, delays in treatment, and replacement or withdrawal of vincristine.^77^

Neuropathic pain is a common complication in cancer patients receiving vincristine chemotherapy, and this phenomenon has initially been modeled in rats.^29^, ^30^, ^31^ Aley and Tanner et al. used male rats to establish a rat neuropathy model by injecting vincristine through the tail vein. Rats were injected intravenously with vincristine 100 μg/kg daily for 2 consecutive weeks, and mechanical hyperalgesia and thermal hyperalgesia occurred. The modeling was preliminarily considered successful. At the same time, Tanner et al. found that vincristine could induce changes such as decreased density of axonal microtubules, disordered arrangement, thickened axonal diameter, axonal swelling, and decreased number of nonmyelinated fibers in peripheral nerves of rats. Next, Nozaki‐Taguchi et al.^31^ modified the animal model originally described to use a continuous infusion technique that eliminated the need for daily intravenous injections for 2 weeks and was considered a more reliable model. Although the exact mechanism of VIPN was not elucidated, further studies using vincristine infusion in this model may help to define common changes in other painful neuropathies. Subsequently, more and more researchers successfully prepared the VIPN model on this basis. Some scholars believe that this model has not been tested in terms of neural aspects, which is a lack of scientific rigor. Therefore, Tanner adopted the same modeling method as before and performed electrophysiological recordings and behavioral tests after 2 weeks of treatment as indicators of successful modeling.^78^ Other studies have shown that intraperitoneal administration causes less neuropathy, lower mortality, less time consumption, and easier operation than intravenous administration. To reduce the suffering of animals, more scholars choose intraperitoneal administration to prepare models. Youn et al.^32^ intraperitoneally administered 100 μg/kg vincristine to Sprague–Dawley (SD) rats for 2 weeks, 5 consecutive days a week, stopped for 2 days, and then continued for 5 consecutive days. This method has been used before.^33^, ^34^ After 2 weeks of treatment, the rats were stimulated with Von‐Frey fibers of different labels to induce mechanical paw withdrawal response, the threshold value (PWT) was >50% and thermal hyperalgesia occurred, which was regarded as a successful model of PN in rats. NCV was not measured. It was also found that different analgesic drugs may provide an alternative approach to the treatment of vincristine‐induced neuropathic pain. Kim et al.^35^ intraperitoneally injected rats with vincristine (100 μg/kg). This leads to the development of cold allodynia, and mechanical and thermal hyperalgesia after 10 days. In addition, an increase in tumor necrosis factor‐α (TNF‐α) was seen in sciatic nerves with neuropathy, suggesting that the inflammatory response may be involved in the development of VIPN. In addition to modeling with rats, some researchers have used Wistar rats for modeling. Considering the dose‐dependent nature of vincristine, Kaur et al.^38^ induced PN by intraperitoneal injection of vincristine at a dose of 50 μg/kg for 10 consecutive days. When vincristine administration was observed to be associated with the development of mechanical hyperalgesia, and heat and cold hyperalgesia after 2 weeks, the VIPN model was considered to be established, and there were no in vivo nerve tests. Muthuraman et al.^39^ injected Wistar rats intraperitoneally with vincristine 75 μg/kg, and regarded the changes in behavior, sciatica function index, and biochemical indexes as successful modeling. Inflammatory cytokines, calcium ion accumulation, and free radical production are thought to play a key role in the neuropathic pain produced by VIPN. Khalilzadeh et al.^40^ also chose adult male Wistar rats to induce neuropathy by intraperitoneal injection of vincristine 100 μg/kg for 2 weeks. The results showed that the body weight decreased significantly on the 7th and 14th day, the latency of tail flapping and mechanical pain threshold decreased significantly after 2 weeks, the sciatic nerve conduction velocity decreased, and pathological changes such as demyelination and degeneration were detected after 2 weeks. The VIPN model was established.

At the same time, domestic and foreign researchers believe that mice can also establish an ideal VIPN model. Babu et al.^41^ induced PN in Swiss Albino mice by intraperitoneal injection of vincristine 100 μg/kg for 7 consecutive days, which was the same as described by Linglu and Katsuyama.^42^, ^43^ After drug administration, mice were tested with a hot plate, cold plate, acupuncture test, rotating rod, and sciatic nerve function index (SFI). The maximum pain threshold was observed on the 7th day, and a significant increase in SFI level was considered as the successful preparation of the VIPN model. Oxidative stress and total calcium levels appeared elevated during administration, and oxidative stress is also thought to be an important factor in the development of PN induced by vincristine in mice, providing a new direction for the treatment of VIPN. No live nerve test results. Next, Bessague et al.^44^ observed that 4–5‐week‐old male Swiss mice were injected with vincristine 100 μg/kg intraperitoneally for 8 consecutive days, and the nerve conduction velocity decreased after 7 days, but not significantly. Immunohistochemical analysis observed decreased nerve fiber density in the mouse foot pad epidermis. Electron microscopy revealed that the sciatic nerve had decreased myelinated fiber density, increased myelinated axon area, disturbed neurofilament and microtubule network, and enlarged microtubules. It suggested obvious peripheral nerve damage, which is consistent with the pathological characteristics of VIPN. VIPN can be induced in Swiss mice, providing a new model for the study of VIPN. Khan et al.^45^ gave vincristine 50 μg/kg to BALB/c mice for 2 consecutive cycles of intraperitoneal injection for 10 days to induce neuropathic pain, which is the same method as previously used in the rat model.^79^ The VIPN mouse model was considered to be successful after significant weight loss, hind limb weakness, panic, and other manifestations, as well as the reduction of mechanical pain, heat pain, and cold pain thresholds in behavioral tests. Lee et al.^46^ used the above method to prepare the model and found that no mice developed any movement disorders or complications, did not gain weight during the treatment period, but had obvious mechanical allodynia after 12 days of treatment, that is, the modeling was considered successful. This may be related to the breed of mice and the dose administered. In addition, immunohistochemical staining found that neurokinin‐1 (NK‐1) and P (SP) substances increased in the spinal cord, which may be related to the mechanism of the inflammatory response caused by VIPN, providing an indicator for studying its pathogenesis. The modeling method of Khan et al.^47^ was similar to that of Gong et al.^48^ and ICR mice were given 75 μg/kg by intraperitoneal injection for 10 days. It was found that there was no significant weight change during the treatment period. Decreased pain thresholds for mechanical allodynia, cold allodynia, and thermal hyperalgesia were observed on behavioral testing. Light microscopy showed loss of myelinated nerve fibers, swelling and irregular arrangement of some axons, and obvious vacuolation in the sciatic nerve. Sciatic nerve conduction velocity was not measured. Gong et al. detected a significant decrease in SNCV and sensory nerve action potential (SNAP) amplitude after administration, suggesting a significant loss of neuronal electrophysiological function, indicating significant PN, and considered the modeling successful. In addition, the study found that the expressions of myeloperoxidase MPO, TNF‐α, and IL‐6 in the spinal cord tissue of mice were significantly upregulated after administration, while IL‐10 played a protective role in VIPN. Vincristine induced an increase in calcium levels. Therefore, inhibition of inflammatory cytokines and accumulation of calcium levels may be beneficial for the recovery from nerve injury.

In conclusion, the model of peripheral neuropathic pain induced by vincristine mainly showed behavioral changes and neuropathological changes in rats and mice, which could well simulate clinical neuropathic pain. It provides a reliable animal model for the study of its mechanism and the use of chemotherapy drugs. This animal model provides a theoretical basis for more effective clinical application of antitumor drugs such as vincristine, and further explores how to prevent and treat complications of PN. It is hoped that a more systematic and comprehensive treatment of vincristine‐induced PN can be achieved in the future.

### 3.3 Platinum‐induced CIPN model

Platinum‐based chemotherapeutics are an antitumor drug, including cisplatin, carboplatin, and OXP, which have always been indispensable in cancer treatment practice and are mainly suitable for the second‐line treatment of malignant lymphoma. Although the clinical indications and pharmacokinetics of these drugs are different, their chemical structures are similar. Platinum‐based chemotherapy drugs generally cannot cross the blood–brain barrier and have little impact on the central nervous system, but their neurotoxic effects can directly affect peripheral nerves. Platinum‐based compounds, including cisplatin and OXP, are usually associated with PN.^80^, ^81^ Clinically, the incidence of peripheral neurotoxicity is higher in patients treated with OXP and cisplatin, and the rate of peripheral nerve injury is 30%–40%.^82^ Carboplatin is much less neurotoxic than cisplatin and OXP.^83^ Currently, PN caused by platinum‐based chemotherapy drugs can only be prevented by reducing the dose or stopping the drug. Therefore, we will summarize the animal model of OXP‐induced PN (OIPN) to further study its pathogenesis and help improve the clinical treatment of patients with OIPN.

OXP is a commonly used first‐line antitumor drug in clinical practice. Like many other anticancer drugs, the treatment causes a common side effect called CIPN, which is the main reason for the drug's dose‐limiting toxicity. Although the basic mechanism of OIPN is not clear, domestic and foreign scholars have not stopped the research in this field. A large number of experiments have been carried out for decades. Next, the research on the CIPN model by domestic and foreign scholars is summarized to provide a reference for subsequent researchers to study the CIPN model.

Zhou et al.^51^ established an OIPN model induced by intraperitoneal injection of OXP 4 mg/kg (2q7d) in male SD rats. During the treatment period, the cold plate test and Von‐Frey filaments stimulated the rats to induce a mechanical foot withdrawal response. It was found that OXP induced significant mechanical pain and cold hyperalgesia, and the OIPN model was considered successful. Hao et al.^52^ constructed the OIPN rat model in the same way. After the treatment, it was found that the model group had significant weight loss, mechanical allodynia, cold and heat hyperalgesia, and severe impairment of motor function, which was regarded as successful modeling. At the same time, immunostaining found that astrocytes and microglia were activated during OIPN induction, suggesting that spinal glial cells may be involved in the initiation and maintenance of OIPN, thereby increasing the pain hypersensitivity response. The modeling method of Li et al.^53^ is similar to that of Yang et al.,^54^ both using male SD rats for intraperitoneal injection. The results showed that the rats in the experimental group started to develop mechanical allodynia and hyperalgesia on the 7th day, and all the rats successfully completed the treatment process without death, indicating that the OXP‐induced OIPN rat model was successfully established, although the nerve conduction velocity was not detected. Zhang et al.^55^ also administered 4 mg/kg (2q7d) intraperitoneally for 4 weeks in SD rats. After 7 days, the body weight decreased significantly. After the treatment, the behaviors showed mechanical pain and cold hyperalgesia, and the conduction velocity of the sciatic nerve was significantly reduced, indicating that the rats had obvious peripheral nerve damage, which was consistent with the pathological characteristics of OIPN, and the modeling was considered successful. This is almost similar to previous experimental studies in rats.^56^, ^57^ In addition, elevated levels of inflammatory cytokines, particularly Interleukin‐1β (IL‐1β) and TNF‐α, contribute to the central sensitization and pain behavior associated with neuropathic pain. In addition to modeling with SD rats, Wistar rats have been used to validate the feasibility of the modeling method by evaluating various physiological indicators and pathological changes. Behavioral and histopathological changes have been detected; therefore, this method can be used for the study of OIPN.^60^, ^61^, ^62^

Likewise, the researchers replicated CIPN in mice. Cold pain and mechanical abnormalities were assessed by hind paw acetone and Von‐Frey. After intraperitoneal injection of OXP 6 mg/kg in C57BL/6 mice, the most significant allodynia signs were observed on the third day and gradually decreased to normal levels in 7–9 days. At the same time, the infiltration of macrophages was induced and the level of IL‐1β in DRG was upregulated, so the OIPN mouse model was successfully prepared.^63^ Rimola et al.^64^ selected male and female C57BL/6NJ mice to study OXP‐induced acute peripheral pain syndrome. After intraperitoneal injection of 3 mg/kg, both male and female mice showed mechanical hypersensitivity at the end of treatment, which was considered as successful modeling. Interestingly, the study found significant changes in the levels of several lipids in neural tissue during OXP‐induced acute pain, yet no changes in chemokines, cytokine levels, or astrocyte activation were observed, suggesting that inflammatory pathways and associated mediators are not as heavily involved in the onset of pain in OIPN as in other chronic pain states, and may only begin to respond secondary to it at a later stage. Ogihara et al.^65^ and Micov et al.^66^ also used C57BL/6 mice to construct an OIPN. Ogihara found that after the treatment, weight loss, peripheral vascular injury, mechanical pain, and abnormality of cold and heat pain occurred, and the detected NCV was significantly reduced, indicating the loss of neurophysiological function, indicating obvious PN. At the same time, the ultrastructure suggested axonal degeneration of the sciatic nerve, and the modeling was further considered successful. Micov also assessed depressive‐like behaviors and motor coordination in mice and found that OIPN mice exhibited loss of motor coordination and depressive‐like behaviors, suggesting peripheral nerve damage. Next, Gould et al.^67^ studied the effect of acute OXP treatment on OIPN in the mouse model. Male C57BL/6Babr mice were selected to evaluate the behavior after a single dose of 6 mg/kg OXP in the peritoneum. The results showed that OXP induced mechanical and cold hypersensitivity reactions, which made the OIPN model. However, Gould found that acute OXP treatment did not induce IENF loss. However, the loss of IENF was found in the OIPN model established by Toyama et al.^68^ Toyama used BALB/c mice to receive an intraperitoneal injection of OXP 10 mg/kg 1q7d for 3 weeks. Behavioral assessments were performed through the development of neuropathic pain and loss of IENF. After the treatment, the mechanical pain and cold pain thresholds of the mice were decreased, and the density of IENF was decreased, which proved the occurrence of PN and suggested the successful establishment of the OIPN model. Similarly, Bouchenaki et al.^69^ used Swiss mice to be intraperitoneally injected with OXP at 15 mg/kg (3 × 5 mg/kg/3 days) to determine whether the modeling was successful by evaluating the behavior, IENF and DRG neuron immunohistochemical analysis, and sciatic nerve ultrastructure. It was found that the mice exhibited transient tactile abnormalities and cold hypersensitivity, unrelated to motor performance or thermal nociception, with no apparent neurodegeneration. This suggests that mimicry of the acute neurotoxicity model does not lead to long‐term degeneration.

In conclusion, the criteria for evaluating the success of OXP‐induced peripheral neuropathic pain models are mainly the presence of behavioral changes and neuropathological changes in rats and mice. In clinical trials, few drugs improved the efficacy of OXP in PN. Therefore, it is very important to develop an in vivo model to further elucidate his mechanism and help improve the clinical treatment of patients with OIPN.

### 3.4 Thalidomide‐induced CIPN model

Thalidomide, a synthetic derivative of glutamic acid, inhibits the production of TNF‐α.^84^ It also has antiangiogenesis and immunomodulatory properties, and can also induce apoptosis of tumor cells.^85^, ^86^ In the treatment of hematological malignancies, it is mainly used in multiple myeloma. At present, it is believed that thalidomide inhibits the development of tumors by inhibiting angiogenesis, stimulating immune system activity, and inhibiting the adhesion of cancer cells to the stroma.^87^ The study found that thalidomide is the first new drug to show significant single‐agent activity for relapsed and refractory multiple myeloma in more than 40 years, and it is often combined with BTZ in clinical treatment.^88^, ^89^, ^90^ Although this new type of drug treatment for multiple myeloma can significantly improve the overall survival of patients, CIPN is the main reason for the reduction or even discontinuation of chemotherapy drugs. It may occur at the end of treatment, and it induces PN that is cumulative, dose‐dependent, and often irreversible.^91^

Previous studies have reported that the incidence of thalidomide‐induced PN (TIPN) is as high as 25%–75%, but the specific mechanism of PN is not yet clear.^92^, ^93^ In hematological malignancies, researchers have given both thalidomide and BTZ to establish an animal model of PN. For example, Liu et al.^70^ established an in vivo model of PN in adult SD rats, using thalidomide administered orally in combination with BTZ subcutaneously or by tail vein injection. At the end of 14 days of treatment, there were signs of systemic toxicity (weight loss) and transmission electron microscopy of the sciatic nerve showed layered structures, tissue breakdown as well as demyelinating changes, which were considered successful in modeling. PN was more severe in combination therapy containing thalidomide compared to therapy in the absence of thalidomide. Thalidomide is prescribed as long‐term maintenance therapy in multiple myeloma. However, due to the lack of existing literature, and thalidomide is mainly combined with BTZ in the treatment of multiple myeloma, there are few animal models of TIPN using thalidomide alone.

## 4 POSSIBLE MECHANISMS OF HEMATOLOGICAL MALIGNANCIES CHEMOTHERAPY DRUGS CAUSING CIPN

### 4.1 Possible mechanisms of BTZ‐induced CIPN

PN caused by BTZ is one of the most common adverse reactions in the clinical treatment of malignant tumors. At present, it is believed that the main pathogenesis of PN caused by BTZ may be as follows.

1. Accumulation in DRG cells: BTZ can accumulate in the DRG neurons in rats and mice and have toxic effects, causing PN. Experiments have confirmed that after adding BTZ to cultured DRG neuron cells, chromatin dissolution occurs in DRG neuron cells, followed by the accumulation of eosinophils in the cytoplasm, resulting in neuronal cell damage and further damage to the nervous system.
2. Damage to mitochondria and endoplasmic reticulum: Inhibition of the 20S proteasome complex by BTZ can activate mitochondria‐dependent apoptotic pathways, resulting in mitochondrial and endoplasmic reticulum damage.^94^, ^95^ Through animal experiments, Cavaletti et al.^13^ observed drug damage to mitochondria and endoplasmic reticulum in DRG, which led to the formation of vacuoles. At the same time, vacuoles appeared in the cytoplasm of satellite cells, and these vacuoles were the damaged mitochondria and endoplasmic reticulum. Therefore, damage to mitochondria and endoplasmic reticulum is the main cause of DRG and sciatic neuropathy.
3. Inhibit the nuclear factor of kappa B (NF‐κB) signaling pathway: The main role of BTZ is to inhibit the activation of nuclear factor NF‐κB, thereby upregulating the proapoptotic protein Noxa and downregulating antiapoptotic target genes, thereby blocking the transcription process of trophic neural factors. So neurotrophic factor dysregulation may play an important role in BIPN.^96^
4. Inflammation: Inflammatory responses play a very important role in neurodegeneration. Some researchers believe that proteasome inhibitors can induce changes in proinflammatory responses and inflammatory processes in neuronal cells, which may be one of the mechanisms of BIPN. Animal experiments have confirmed that TNF‐α is involved in BIPN, application of BTZ can upregulate the expression of TNF‐α in DRG neurons, and TNF‐α can also increase the level of heparinase (HPSE), thereby triggering TNF‐α and the release of other inflammatory cytokines, eventually inducing BIPN.^16^
5. Other factors: Some autoimmune factors, defects in the axonal transport of tubulin, and genetic factors can also contribute to the occurrence of PN induced by BTZ, but the specific mechanism has been further studied.

### 4.2 Possible mechanisms of vincristine‐induced CIPN

At present, it is believed that the main pathogenesis of PN caused by vincristine treatment is as follows.

1. Regulation of opioid‐related receptors: Vincristine‐induced CIPN is associated with a reduction in endomorphin‐2 levels, which disrupts its pain‐relieving effects on mu‐opioid receptors, leading to hypersensitivity of C‐fiber nociceptors and the development of CIPN.^36^
2. Changes in calcium ion movement in mitochondria: Vincristine affects the movement of calcium ions through the mitochondrial membrane, maximizing calcium ion storage and reducing its efflux. Alterations in the movement of calcium ions in mitochondria can affect calcium movement across the mitochondrial membrane of DRG neurons, altering homeostasis and leading to abnormal neuronal excitability.^50^
3. Inflammatory response: Vincristine attracts immune cells and activates the release and elevation of proinflammatory cytokines (interleukins and chemokines), leading to neuroinflammation.^49^
4. Alterations in spinal synaptic plasticity that sustain neurological symptoms: Expression of C‐Fos (a marker of neuronal activation) and Picclio (maintaining synaptic plasticity) were increased in spinal cord neurons in an animal model of CIPN. These results indicate that neuronal activity is increased and the presynaptic elements are restructured.^37^

### 4.3 Possible mechanisms of platinum‐induced CIPN

At present, it is believed that the main pathogenesis of CIPN caused by platinum compounds may have the following points.

1. Damage to nuclear DNA and mitochondrial DNA: Platinum‐based chemotherapeutics are alkylating drugs that can cause nuclear damage and induce DNA cross‐linking to form platinum‐DNA conjugates. It leads to mitochondrial damage, affects the replication process, and arrests the cell cycle, eventually causing apoptosis and death of neuronal cells.^97^
2. Oxidative stress: It is well known that mitochondrial dysfunction induces cellular oxidative stress. Cisplatin treatment can produce the release of reactive oxygen species (ROS), resulting in oxidative damage to cells, including DRG neurons, which are the main site of drug accumulation, resulting in neuronal cell damage.^98^
3. Disorders of ion channels: Ion channels are also toxic targets of platinum‐based chemotherapeutics. Calcium dysregulation can induce abnormal spontaneous firing of neuronal cells, neurofibrillary degeneration, and central sensitization, promoting allodynia and hyperalgesia.^58^ Studies have also shown that administration of OXP can induce hyperexcitability of neurons in mice, reduce the expression of potassium ion channel, double‐porous potassium ion channel TREK‐1, and protein TRAAK, and increase the hyperpolarized cyclic nucleosides in DRG neurons and acid‐gated channels, causing neurotoxicity.^59^

### 4.4 Possible mechanisms of thalidomide‐induced CIPN

TIPN is a sensory axonal polyneuropathy that usually occurs in patients with severe sensory symptoms. However, the mechanism of TIPN has not been elucidated. At present, it is believed that the main pathogenesis of PN caused by thalidomide treatment may have the following points.

1. Antiangiogenesis: When thalidomide exerts its antitumor effect, it blocks angiogenesis by inhibiting the basic fibroblast growth factor and vascular endothelial growth factor, and inhibits the formation of new blood vessels around peripheral nerves, reducing the blood supply to nerve fibers and making nerve fibers. Demyelinating lesions occur due to ischemia and hypoxia.^99^
2. Block the activation of NF‐κB: Thalidomide can downregulate the expression of NF‐κB and block the growth effect of neurotrophic factors, thereby leading to abnormal growth regulation of peripheral nerves or direct toxic effects on neuronal DRG, resulting in DRG degeneration, which leads to the occurrence of PN.^100^
3. Genetic factors: Some studies have also suggested that genetic variation may be involved in the neurotoxicity of drugs, leading to the occurrence of PN.^101^

## 5 TREATMENT OF CIPN AFTER CHEMOTHERAPY FOR HEMATOLOGICAL MALIGNANCIES

In view of the high incidence of CIPN in the process of antitumor treatment, and the dose‐limiting toxicity, the use of neuroprotective drugs is of great significance to prevent this complication. Neuroprotective drugs including duloxetine, amifostine, B vitamins, antioxidants such as vitamin E, alpha‐lipoic acid, glutathione and glutamine, and minocycline have certain effects on the prevention and treatment of CIPN. However, evidence‐based medicine is insufficient, and it can be used clinically as appropriate.^102^, ^103^, ^104^ The American Society of Clinical Oncology (ASCO) guidelines states that duloxetine as a serotonin–norepinephrine reuptake inhibitor is the only recommended treatment.^6^ Neurotropine has been reported to reverse paclitaxel‐ and OXP‐induced CIPN in rats.^105^, ^106^ An animal study showed that neurotropin alleviates OXP‐induced CIPN by inhibiting axonal degeneration and may have clinical potential for the treatment of OXP‐induced neuropathy.^107^ In addition, once PN occurs in the treatment of hematological malignancies, in addition to adjusting the drug dose or interrupting the treatment, it is necessary to reduce the severity of its neurological symptoms. Currently, for the management of neuropathic pain, multimodal analgesia is recommended, including opioids (morphine or codeine, etc.), antiepileptic drugs (gabapentin, pregabalin, etc.) and some antidepressants (amitriptyline, venfalacine, or imipramine, etc.) ^4^, ^5^, ^108^, ^109^

Traditional Chinese medicine therapies including gastrodin, Astragalus Guizhi Wuwu Decoction, and acupuncture, are also widely used to treat CIPN. Qin et al.^110^ found that gastrodin administration could alleviate chronic pain in a rat CIPN model and attenuate inflammation by reducing the expression of TNF‐α and IL‐6. Through animal studies, Cheng et al.^61^ found in animal studies that Huangqi Guizhi Wuwu granules had a reparative effect on OXP‐induced DRG damage and could prevent OXP‐induced neuropathy without reducing its antitumor activity. Lv et al.^111^ also found that Huangqi Guizhi Wuwu decoction could reduce the incidence of CIPN and inhibit paclitaxel‐induced inflammation and oxidative response in the peripheral nervous system. Studies have shown that acupuncture and moxibustion in the prevention and treatment of CIPN have the advantages of low cost, safety, and simple operation. Many researchers have found that acupuncture can effectively improve pain and quality of life in CIPN.^112^, ^113^, ^114^ Han et al.^115^ found that acupuncture combined with mecobalamin can significantly improve CIPN‐related pain, which is significantly better than mecobalamin alone, and nerve conduction velocity is significantly improved. Although these trials have been reported for the treatment of CIPN, the options for drugs that can effectively reduce or treat CIPN are limited.

## 6 CONCLUSIONS AND FUTURE PERSPECTIVES

In conclusion, chemotherapy is still the first choice for the treatment of many hematological malignancies. However, in the course of clinical treatment, the pathogenesis, incidence, symptoms, and severity of PN caused by different chemotherapeutic drugs are different, and different drug‐specific methods may be required to prevent the development of nerve damage. In recent decades, with the use of novel targeted drugs for the treatment of hematological malignancies, the incidence of CIPN has gradually increased. CIPN can occur during and after treatment with BTZ, thalidomide, vincristine, cisplatin, and OXP. Therefore, early identification of PN is critical. Fortunately, second‐generation proteasome inhibitors, such as carfilzomib, and immunomodulators, such as lenalidomide and pomalidomide, reduce the risk of PN. Despite the development of drugs associated with reducing toxicity, options for the treatment and prevention of CIPN remain limited.

The construction of experimental animal models is a necessary way to study the mechanism of CIPN and its prevention and treatment. Existing CIPN animal models, such as BIPN, VIPN, and OIPN, do not affect the antitumor activity of chemotherapeutic drugs themselves and seem to be more suitable for the study of CIPN. However, mouse models of CIPN do not well mimic the tumor environment of a patient, and in the future, the use of efficient and robust animal models that mimics the clinical situation is needed; CIPN models that mimic the hematological malignancies type will be improved, which will provide experimental support for clinically alleviating the adverse effects of chemotherapy and the suffering of patients.

## AUTHOR CONTRIBUTIONS

Xiaoli Lv, Yingwei Mao, and Yonghuai Feng contributed to the main ideas of this review, leading to the submission of the paper and the collection of resources. Song Cao contributed to the organizational thinking and revision of the paper, finalized the manuscript, and approved the final version for review.

## CONFLICT OF INTEREST

Song Cao is the editorial member of Ibrain, who has not been involved in the peer review process. 

## ETHICS STATEMENT

There are no possible animal or medical ethical issues for this review article.

## Data Availability

Data reported in this study are available from the Lead Contact on request.
